# Diagnostic and medical needs for therapeutic drug monitoring of antibiotics

**DOI:** 10.1007/s10096-019-03769-8

**Published:** 2019-12-11

**Authors:** Claude Mabilat, Marie Francoise Gros, David Nicolau, Johan W. Mouton, Julien Textoris, Jason A. Roberts, Menino O. Cotta, Alex van Belkum, Isabelle Caniaux

**Affiliations:** 1 Medical Affairs, bioMérieux, Marcy l’Étoile, Lyon, France; 2 Center for Anti-Infective Research & Development, Hartford Hospital, 80 Seymour Street, Hartford, CT 06102 USA; 3 Department of Medical Microbiology and Infectious Diseases, Erasmus University Medical Centre, Rotterdam, Dr Molewaterplein 40, 3015 GD Rotterdam, Netherlands; 4 Centre for Clinical Research, Faculty of Medicine, The University of Queensland, Brisbane, Queensland Australia; 5 School of Pharmacy, The University of Queensland, Brisbane, Queensland Australia; 6 Pharmacy Department, Royal Brisbane and Women’s Hospital, Brisbane, Queensland Australia; 7 Data Analytics Department, bioMérieux, La Balme Les Grottes, Grenoble, France

## Abstract

Therapeutic drug monitoring (TDM) of antibiotics has been practiced for more than half a century, but it is still not widely applied for infected patients. It has a traditional focus on limiting toxicity of specific classes of antibiotics such as aminoglycosides and vancomycin. With more patients in critical care with higher levels of sickness severity and immunosuppression as well as an increasingly obese and ageing population, an increasing risk of suboptimal antibiotic exposure continues to escalate. As such, the value of TDM continues to expand, especially for beta-lactams which constitute the most frequently used antibiotic class. To date, the minimum inhibitory concentration (MIC) of infectious microbes rather than classification in terms of susceptible and resistant can be reported. In parallel, increasingly sophisticated TDM technology is becoming available ensuring that TDM is feasible and can deliver personalized antibiotic dosing schemes. There is an obvious need for extensive studies that will quantify the improvements in clinical outcome of individual TDM-guided dosing. We suggest that a broad diagnostic and medical investigation of the TDM arena, including market analyses and analytical technology assessment, is a current priority.

## Introduction

The need for precise measurement of antimicrobial agents in the blood of patients to follow treatment success or failure was recognized early on [1]. Traditionally, this therapeutic drug monitoring (TDM) of antibiotics has primarily involved quantitative drug measurements in patient’s plasma to minimize toxicity risks with agents of narrow therapeutic indices [2, 3]. Still for antibiotics, TDM was used sparingly such as for vancomycin, chloramphenicol and the aminoglycosides [4]. The general target population were and still are patients in acute care situations or those hospitalized for longer term care and treatment (e.g. [5]). A more advanced TDM practice, however, is emerging with an aim to ensure efficacy as well as minimizing toxicity of a broader spectrum of antibiotics with relatively wider therapeutic indices. It takes advantage of a better definition of the optimum antibiotic exposure for therapeutic success. Antibiotic pharmacokinetics (PK) is commonly defined as ‘what does the body do to the drug’ during its complete cycle in vivo, whereas pharmacodynamics (PD) relates to ‘what the drug does to the microorganism *in vivo’*, the interplay between the two, ‘PK/PD’, defining the optimum antimicrobial activity achievable for the unbound drug concentrations at the site of infection. Consensus studies and guidelines have helped to propose values for these PK/PD targets, specific for an antibiotic in combination with a clinical condition [6–8]. By comparing drug plasma concentration data (a surrogate for the site of infection) to these predefined PK/PD targets, a personalized antibiotic dosing practice can help ensure an optimized antibiotic exposure. Personalized treatment will apply to an increasing population of high-risk patients and will have additional impact on the fight against antimicrobial resistance (AMR) by significantly improving the optimal usage of our pharmacopoeia. Finally, a reduced developmental pipeline for new antibiotics is necessitating that we spare last resort drugs with TDM considered instrumental for protecting this precious antimicrobial armamentarium (e.g. [9]).

Here, we succinctly review the current status of TDM in the field of antibiotic therapy. Despite the fact that significant progress has been made in various disciplines in the field of TDM, there still seems to be a restricted global implementation.

## Sub-optimal antibiotic exposure in high-risk patient groups

The majority of drug dosing regimens has been defined in healthy adults at the time of drug development where there was no resistance to those drugs. The lack of variability in PK and patient characteristics in those initial studies is not a representative of the breadth of patients in whom these drugs will be used and the ultimate exposure levels of such patients. It is highly unlikely that this “one dose fits all” strategy will maximize antibiotic effectiveness in all patients. Unpredictable drug concentrations in plasma and other body compartments due to altered volume of distribution and clearance may result from factors as diverse as acute pathophysiology during critical care admission (such as sepsis and immuno-suppression), obesity [10], early ages [11], advanced age, surgical prophylaxis for longer invasive procedures [12], cystic fibrosis [13], the use of techniques for organ replacement in case of organ failure (extracorporeal membrane oxygenation (ECMO)) and renal replacement therapy (RRT) [14, 15]. To add to these significant alterations of PK in critically ill patients, the PD target might be different depending on the indications. (e.g. skin and soft tissue versus pulmonary infections).

As a consequence, a growing body of evidence links sub-therapeutic antibiotic concentrations with treatment failure. For example, the “Defining Antibiotic Levels in Intensive Care Unit (ICU) patients” (DALI) study showed that up to 70% of critically ill patients in the ICU did not meet the PK/PD targets defined for beta-lactams, and that those patients with the lowest exposures were more likely to have a negative treatment outcome (treatment course with change or addition of antibiotic therapy, in particular within 48 h of cessation) [16]. Similar data are available for other antibiotics including meropenem [17], other beta-lactams [18–20], vancomycin [21–23], and ciprofloxacin [22, 24]. Both the studies by Moise-Broder et al. [21] and Martirosov et al. [23] show significant differences between optimal and suboptimal treatment of staphylococcal infection where the area under the curve (AUC) for 24 h of treatment below or under the minimum inhibitory concentration (MIC) value is statistically significant. The combined analysis of 179 patients clearly show that optimal vancomycin dosing is an important denominator of clinical and microbiological success. The use and therapeutic success of intravenous ciprofloxacin in 74 patients was shown to be dosing dependent [24]. Low dosing at 200 mg for every 12 h versus higher dosing at 400 mg every 8 h resulted in different overall drug concentrations in the blood and were clearly associated with slower eradication of the infecting organism in the low concentration treatment group. Of note, additional patient’ characteristics have a distinct effect on antibiotic concentration in the circulation. Carrie et al. [20] show that augmented renal clearance of beta-lactams resulted in suboptimal antibiotic concentrations in serum for 12% of the patients and with that a significant effect on clinical and bacterial cure. Sub-optimal dosing and its clinical consequences is a domain of important research that should be thoroughly included in any development protocol for new antimicrobials.

## Optimized dosing for reducing selection of resistance

The decreasing antibiotic susceptibility of pathogens may require higher antimicrobial dosing to achieve a therapeutic exposure that maximizes treatment success [25]. Still, dosing regimens for a new drug are initially defined by targeting ‘wild type’ susceptible pathogens, from which are derived the so-called ecological cut-off values (ECOFF) (MIC distribution separating bacterial populations into wild type and those with acquired or mutational resistance, see for instance [26] or the European Committee for Antimicrobial Susceptibility Testing (EUCAST) definition of wild type bacterial isolates). When a non-wild type pathogen emerges, higher doses may have to be used even if its higher MIC still falls within the susceptible or in the intermediate categories. As physicians tend to exclude antibiotics associated with intermediate MICs in favour of alternative antibiotics for which the bacterial strain is still fully susceptible, potent broad spectrum antimicrobials are often used [27]. Within this context, the EUCAST and Clinical and Laboratory Standards Institute (CLSI) committees recently promoted new increased dosing regimen categories in order to help spare last resort antibiotics (see EUCAST (www.eucast.org) and CLSI (www.clsi.org) websites for more detail). Thus, the dynamics of what the antibiotic does to the infectious bacteria in vivo has to be revised according to the MIC of the strain. EUCAST representatives suggest to integrate inherent experimental variability of MIC testing into the calculation of PK/PD targets by applying the following rules [28]. First, if the MIC is in the wild type category (susceptible organism) and lower than the ECOFF, then the ECOFF value should be used as the MIC value in the calculation of the PK/PD index. Second, if the MIC is above the ECOFF, then a ×4 MIC value should be used. Thus, stricter adherence to antimicrobial susceptibility testing (AST) guidelines will make the data generated by TDM even more clinically useful. It has to be acknowledged though that MICs represent a marker based on a single point in time and concentration. In addition, measuring an MIC is no sinecure and significant variability within and between methods exists [29]. Not everything will be solved by simple adherence to guidelines, and there is a need for further studies into optimized dosing as a consequence of regional prevalence of antibiotic resistance [30].

## Patient outcomes and PK/PD target attainment

An increased body of knowledge provides evidence for benefits associated with PK/PD target attainment, including variations in the dosing schemes, in terms of lower mortality, clinical cure, reduced length of stay and lower toxicity, as shown for many antibiotics. For example, continuous infusion as opposed to short-term infusion can help to reach more effective PK/PD targets and improve efficacy in case of sepsis where mortality can be reduced up to 30% [31, 32]. Vancomycin [33, 34], beta-lactams [35, 36], aminoglycosides [37, 38], quinolones [24, 39], linezolid [40], daptomycin [41] and even antifungals [42] have been studied in adequate detail although it has to be admitted that not all studies uniformly showed clinical benefit.

Few studies have evaluated the impact of active TDM per se on the clinical course of critical care patients in prospective randomized trials [43], except maybe in the on-going “DIABOLO” and “OPTIMAL TDM” clinical trials [44]. These studies show that TDM improves antibiotic exposure by guiding PK/PD target attainment. This will potentially help reduce the early development of antibiotic resistance as evidenced in preclinical infection models [45, 46] and adds weight to the call for active TDM of all patients, for which high PK variability is expected. This is corroborated by Jacobs et al. [47] who showed that a simple creatinine clearance measure cannot be used to predict and adjust the beta-lactam plasma concentration in septic patients with augmented renal clearance [48]. Independently, TDM was proven to be cost-effective for streamlining aminoglycoside use [49]. This can only be true for newer, hence more costly antibiotics. Thus, it can be concluded that, if managed correctly, TDM is profitable for patient management and the health economy simultaneously.

## Monitoring of antibiotic concentrations today

From a historical perspective, TDM has been adopted mostly to prevent adverse toxicity effects, mainly for glycopeptides and aminoglycosides. Apart from the assessment of trough concentrations, also the determination of peak concentrations was considered to be diagnostically and, hence, therapeutically important. TDM of trough concentrations still is strongly recommended for all patients using the aforementioned antibiotics [17, 50].

However, TDM is now on a slow rise in broader use to ensure optimum antibiotic exposure, in particular to avoid under-dosing, with evidence existing for many antibiotic classes including beta-lactams, linezolid and daptomycin (see statements above). With regard to beta-lactams, the most commonly monitored drugs are piperacillin-tazobactam and meropenem, followed by ceftazidime [51]. Data required include the concentration of the antibiotic in the patient’s plasma, which is quantified either by a specialized pharmacology or biochemistry laboratory, as well as the MIC of the infectious microorganism, a result originating from the microbiology lab. These data are then consolidated with other clinical parameters (patient weight, comorbidities, renal function, etc.) TDM may be managed by routine hospital laboratories or more specialized independent laboratories either or not “Clinical Laboratory Improvement Amendments” (CLIA) compatible or waived.

## Technologies used for TDM

The most commonly used method for current TDM involves immunological assaying, mostly using closed systems and automated chains. These are used in commercial kits for ‘toxic’ antibiotics like vancomycin and aminoglycosides and are mostly appreciated for their rapidity. However, standardization and calibration of this type of testing is not always easy. Immunoassays are often adapted to open chemistry mainstream chains to lower the overall cost (e.g. CEDIA by ThermoFisher Scientific and Roche, enzyme multiplied immunoassay technique (EMIT) by Olympus Chemistry Systems and the tests provided by Randox Laboratories (Crumlin, UK)).

The second most used technology is that of liquid chromatography (LC) which is often combined with mass spectrometry (LC-MS). LC-MS uses mostly locally developed assays and protocols, which provides a level of experimental flexibility that can be easily adapted to commonly applied antibiotics. LC-MS is a highly adaptable technology, and when internal standard molecular markers are available and the equipment and associated personnel are within financial reach, this is a good diagnostic choice. On the other hand, duration of MS technologies (24–96 h) may pose a significant obstacle to clinical use. Application of LC-MS requires the employment of an expert scientist which makes a 24-h service difficult. The technology is not available in all laboratories but mainly in metropolitan-based laboratories which often leads to delayed or prolonged time to result. Actually, various forms of point of care testing are currently being developed and prototypes are being experimented with. The use of biosensor technologies has been proposed, superficially explored and the first results are promising [52–54]. In the latter study, elegant microneedle-based biosensors were used for beta-lactam detection. Recent publications revealed the successful use of microfluidic devices for monitoring aminoglycoside or beta-lactam concentrations in whole blood with a wide dynamic range [55, 56]. The development of such miniaturized and potentially automatable methods can better position TDM in routine microbiology laboratories, particularly if they are shown to be cost-effective. The assaying of body fluids that can be obtained non-invasively may make TDM more feasible [57]. The major current need are large-scale clinical validation studies for these new technologies. As stated before: none of the currently available methods adequately distinguished between the bound and unbound fractions of antibiotics.

## Clinical timing of TDM

It is suggested that for clinical application of TDM, one or two TDM measurements should be made during the very first dosing interval of the antibiotic treatment, then updated as much as needed after 48 h or before, depending on the antibiotic. The data can then be compared with PK models for specific populations, now available in any one of various software solutions that estimate the dose to use [58]. In practice, this is difficult because antibiotics might have short half-lives (4 h and 6 h on average for β-lactams and vancomycin, respectively). Defining the best sampling time may be problematic since the prescription strategy often relies on repeated antibiotic administration. This underlines the need for well-documented, written protocols for managing TDM. Another challenge is that the total turnaround time (TAT) of TDM may be too long if the required expertise is not available as a permanent service. This includes the need for sometimes complicated and lasting pre- and post-analytical procedures; total TDM TAT ranges between 2 h to sometimes even 24 h, averaging 18–24 h (personal communications with various TDM users). With the actual TDM test being relatively quick in itself (ca. 30 min), the overall TAT is limited by logistics and pre- and post-analytical processes. This underscores the need for a simple-to-operate and easy-to-interpret test that must be continuously available to help increase the operability of TDM.

Of additional interest is the previously already mentioned use of continuous infusion of antibiotics in the ICU [31, 59]. The first intelligent devices that can perform this in an intrinsically controlled fashion using concentration-based feedback mechanisms are becoming tested in this field [60]. This also translates into a considerable practical interest for TDM because once the initial loading dose has attained its equilibrium, sampling and TDM can be done at any time, therefore removing time constraint and kinetic uncertainty and enabling a smoother use of TDM in the patient management workflow [61]. In addition, the use of machine learning procedures to develop algorithms that facilitate software-driven timing-independent adaptation of individual treatment will become more widespread in the near future.

## Conclusion and recommendation

TDM should be clearly positioned as an integral part of the antimicrobial stewardship program in hospitals given its pivotal role in antibiotic treatment optimization. This will enable better delivery of the right antibiotic dose for the right patient at the right moment and for the right duration. As such, it will help combat the challenges provided by the growing AMR problem. This personalized medicine perspective still requires improvement to fully satisfy antibiotic prescribers. It is urgent that the TAT of TDM be shortened significantly and that accessible point of care tests and rapid technology–based assays enter the TDM market. Also, here are many practical questions around the implementation of TDM. How to coordinate results coming from microbiology and clinical chemistry laboratories? Who will integrate the data and issue a therapeutic advice? Who will be adjusting antimicrobial therapy and along which guidelines? When these issues have been resolved, TDM should become an integral part of the standard testing portfolio of all self-respecting clinical microbiology laboratories.
